# HIV misdiagnosis in paediatrics: unpacking the complexity

**DOI:** 10.7448/IAS.20.7.21959

**Published:** 2017-08-29

**Authors:** Emma Sacks, Jennifer Cohn, Martina Penazzato

**Affiliations:** ^a^ Department of Research, Elizabeth Glaser Pediatric AIDS Foundation, Washington, DC, USA​; ^b^ Department of Innovation and New Technology, Elizabeth Glaser Pediatric AIDS Foundation, Geneva, Switzerland; ^c^ Division of Infectious Diseases, University of Pennsylvania School of Medicine, Philadelphia, PA, USA; ^d^ HIV Department, World Health Organization, Geneva, Switzerland

## Introduction

Timely and accurate diagnosis of paediatric HIV infection continues to be a public health challenge. Scale up of interventions to prevent mother-to-child HIV transmission (PMTCT) has facilitated the strengthening of early infant diagnosis (EID) programmes, but more needs to be done to ensure that infants and children infected with HIV are identified in a timely manner and effectively linked to care and treatment. In particular, HIV testing of those HIV-exposed children missed by PMTCT services will require more focused attention and increasingly more innovative approaches. In addition to optimizing the appropriate use of HIV testing along the paediatric diagnostic cascade, it is critical to minimize misdiagnosis to prevent mortality resulting from false negative testing, unnecessary lifelong treatment in HIV-uninfected children who receive a false positive diagnosis and potential psychosocial sequelae resulting from misdiagnosis.

While it is true that children face many of the same issues with misdiagnosis as adults, including the accuracy of currently available technologies, cross-contamination or mislabelling of specimens, inadequate adherence to testing guidelines and algorithms, and issues with interpretation of test results, a number of issues specific to paediatric testing need to be considered. Transmission dynamics, natural history and decay of maternal antibodies result in additional complexities that may affect interpretation and management of test results. Both virological and serological test performance may also be affected by the timing of testing and exposure to antiretroviral (ARV) drugs taken by the mother and/or the infant. Overall, the rapid progression of disease among untreated, infected children further highlights the importance of minimizing misdiagnosis and ensuring prompt identification of HIV-infected infants and children.

## Unique challenges of HIV testing in infants and children

There are a number of challenges across the paediatric HIV diagnostic cascade for children which relate to the use of virological or antibody testing for the purpose of assessing HIV exposure and/or identifying HIV infection (Table 1​). Virological testing is required to ascertain HIV status in infants and children below 18 months. A number of molecular testing platforms are currently available for EID, including conventional testing and the newer point-of-care (POC) platforms. Misdiagnosis, with false negatives, false positives and indeterminate results, has been reported in a number of settings [1–3]. False negative results are of greatest concern in terms of risk to the infant but little is known about the implications of indeterminate results. The performance of these nucleic acid tests (NATs) varies and, especially in infants, could depend on a number of factors. Transmission and viral dynamics are the most important, particularly as more effective PMTCT interventions are being scaled up. Timing of testing plays an obvious but important role: intrauterine transmissions can be identified with virological testing as early as birth, while intrapartum and early breastfeeding transmissions require NAT at a later point, usually at age 4–6 weeks.

**Table 1.** Potential issues and mitigating actions for misdiagnosis in paediatric HIV testing​

	Potential issues	Mitigating actions
*Falsepositives*		
NAT	Reduction in positive predictive value with decreasing transmission rates	Confirmatory testing upon initial positive NAT (with conventional or POC NAT)
Antibodies	Patterns of maternal antibody decay and infant antibodies production	Ensuring appropriate timing and interpretation of RDT results by health care workers​
*False negatives*		
NAT	Transmission dynamic and timing of infectionExposure to ARV (particularly enhanced prophylaxis)	Ensure implementation of WHO testing algorithmEnsuring ascertainment of final diagnosis at the end of breastfeedingGather additional data on impact of ARV exposure on NAT results
Antibodies	Patterns of maternal ab decay and infant antibodies productionImmunological suppression resulting in lack of antibody responseSeroreversion as a result of early ART initiation	Ensure implementation of WHO testing algorithmEnsuring ascertainment of final diagnosis at the end of breastfeedingNo repeat testing once diagnosis is confirmed and ART is startedGather evidence on false negative RDT for asymptomatic infants with severe immunosuppression

Exposure to maternal ARV or postnatal prophylaxis (particularly when multi-drug or prolonged) is another potential factor that may reduce the viral load in an infected infant and lower the sensitivity of the test. However, to date, evidence is sparse and conflicting, with some studies documenting a delay in detectability [1,4] and others confirming identification of virus load as low as 40 copies/ml [5], even when POC assays are used. Evidence generated after the WHO guidelines were issued demonstrate good performance at birth, as well as 6 weeks, with sensitivity and specificity ranging from 93.3–100% and 99–100%, respectively [5–10]. As suggested by Technau et al. in this supplement, exposure to ARVs may also increase the number of indeterminate results (defined as a detected target with a cycle threshold (Ct) greater than 33 on a quantitative PCR) and these infants are often diagnosed as HIV-infected at later testing [11]. These factors make it critical that infants with negative NAT are retained in the postnatal period and offered later testing to fully rule out infection as well as to offer continued support in preventing transmission through breast milk.

Use of rapid antibody diagnostic tests (RDTs) presents a separate set of challenges regarding the potential for paediatric misdiagnosis. RDTs are used both to document HIV exposure in infants as well as to diagnose HIV after 18 months of age. A recent review that informed WHO guidelines highlights the potential for false negative results when infants are tested for exposure after 4 months, with sensitivity as low as 60% [12]. This phenomenon results from the decay of maternal antibodies below the threshold of detectability, highlighting the importance of testing the mother whenever possible or using cautious interpretation of any negative results in infants tested after 4 months whose mother’s status is unknown. Use of RDT for diagnosis requires infant production of antibodies, which relates to time of transmission and may be attenuated in infants that are already significantly immunosuppressed as a result of HIV infection. While a negative RDT after 9 months is considered reliable to exclude current HIV infection, it does not provide final diagnosis. This is particularly true for infants who are ill and who may have a false negative RDT, and for those still at risk due to breastfeeding [13]. Similar to NAT, repeat longitudinal testing is critical to ensure that risk of misdiagnosis is minimized.

## Evolving scenarios and new issues

Scale up of PMTCT and early ART for infants and children will likely add additional layers of complexity. First, as PMTCT decreases perinatal transmission, the positive predictive value of a single test will continue to decrease, resulting in potentially more false positive results, highlighting the importance of ensuring confirmatory testing is provided. Recent cost-effective analysis indicates that confirmatory testing is cost-effective and its value increases as transmission rates decrease [14]. However, repeat testing should not delay ART initiation and treatment should be started upon the first positive result. POC may be useful in providing a more rapid confirmation of a positive initial test.

POC NAT is a new technology and its benefits over conventional testing, including more rapid turnaround time and increased percentage of HIV-infected children initiated on ART, suggest that, at current prices, it may be cost-effective as compared to conventional NAT [15]. Studies have begun to formally assess the cost-effectiveness of POC versus conventional NAT for EID. Additionally, the possibility of cost containment with bulk purchasing of equipment and materials, multiplexing (for EID, viral load and tuberculosis), and the creation of testing and transport networks to share machines, these platforms may be shown to be a worthwhile investment.

Second, children that are started on treatment early in life may never develop HIV antibodies and have false negative results on RDT as shown in Ferrand in this supplement [16]. A number of studies have documented that this phenomenon is more likely when infants are started on ART in the first months of life [17,18], but false negatives may also occur when ART is started later, particularly when oral RDTs are used. This has the potential to generate confusion among providers and the family and may present the dilemma of interrupting ART to see if the child is truly infected. While NAT may help to resolve some of these cases, false negative results may also occur with NAT especially if the child is fully suppressed on ART [19]. Therefore, ensuring that an accurate diagnosis is conducted with specimens collected before ART is started will be critical. This will also require appropriate messaging to caregivers regarding the potential for false negatives in children treated with early ART.

Overall, there is almost no qualitative research on issues regarding misdiagnosis of HIV testing in children. A paucity of information exists about caregivers’ understanding of discordant results, barriers to or information needed for informed decision-making about potential lifetime treatment initiation for infants, and the social consequences, including issues around disclosure to family and friends, of indeterminate and delayed results. Little is understood about practical or behavioural implications of changing diagnoses for families who are affected, but even with limited evidence, given historical mistrust around HIV, programmes should be especially sensitive to the potential for misdiagnosis to erode trust between families and the health system and should proactively express the possibilities and mitigation strategies for misdiagnosis to all involved.

## Conclusions

In the current HIV response, the primary issue in paediatric HIV diagnosis is ensuring the scale up of timely HIV testing in infants and children, but misdiagnosis should not be forgotten. The causes of misdiagnosis in children are complex, yet there is reason for optimism. Complex viral dynamics, coupled with the high mortality for untreated, HIV-infected infants, make it even more critical to ensure children complete the entire diagnostic cascade, providing multiple opportunities to diagnosis HIV-infected children. New developments in diagnostic technologies, such as POC NAT, are changing the landscape and improving timely patient access to appropriate diagnostic modalities. Strategies to improve accuracy of diagnosis, as well as timely receipt of results and follow-up, are paramount to improving care and reducing HIV-related mortality that disproportionally affects HIV-exposed infants.

In summary, key actions to minimize misdiagnosis include (1) ensuring that all HIV-exposed infants and children are retained and complete the WHO-recommended testing cascade until final diagnosis is ascertained after completion of breastfeeding; (2) confirmatory testing is provided to any child who has a positive initial NAT; (3) once diagnosis is confirmed and ART is started, further testing is not recommended and, if conducted, negative results should be interpreted with extreme caution; and (4) clear messaging and community awareness about the importance of the entire EID cascade is critical. These actions need to be considered in the context of a more strategic integration of paediatric HIV testing into the wider child survival platform to mainstream and expand access to quality and timely HIV testing.
